# Light-Dependant Biostabilisation of Sediments by Stromatolite Assemblages

**DOI:** 10.1371/journal.pone.0003176

**Published:** 2008-09-10

**Authors:** David M. Paterson, Rebecca J. Aspden, Pieter T. Visscher, Mireille Consalvey, Miriam S. Andres, Alan W. Decho, John Stolz, R. Pamela Reid

**Affiliations:** 1 Sediment Ecology Research Group, Gatty Marine Laboratory, School of Biology, University of St Andrews, St Andrews, United Kingdom; 2 Department of Marine Sciences, University of Connecticut, Groton, Conneticut, United States of America; 3 National Institute of Water and Atmospheric Research Ltd, Greta Point, Wellington, New Zealand; 4 Rosenstiel School of Marine and Atmospheric Science, Division of Marine Geology and Geophysics, University of Miami, Miami, Florida, United States of America; 5 Department of Environmental Health Science, Arnold School of Public Health, University of South Carolina Columbia, Columbia, South Carolina, United States of America; 6 Department of Biological Sciences, Duquesne University, Pittsburgh, Pennsylvania, United States of America; University of Sheffield, United Kingdom

## Abstract

For the first time we have investigated the natural ecosystem engineering capacity of stromatolitic microbial assemblages. Stromatolites are laminated sedimentary structures formed by microbial activity and are considered to have dominated the shallows of the Precambrian oceans. Their fossilised remains are the most ancient unambiguous record of early life on earth. Stromatolites can therefore be considered as the first recognisable ecosystems on the planet. However, while many discussions have taken place over their structure and form, we have very little information on their functional ecology and how such assemblages persisted despite strong eternal forcing from wind and waves. The capture and binding of sediment is clearly a critical feature for the formation and persistence of stromatolite assemblages. Here, we investigated the ecosystem engineering capacity of stromatolitic microbial assemblages with respect to their ability to stabilise sediment using material from one of the few remaining living stromatolite systems (Highborne Cay, Bahamas). It was shown that the most effective assemblages could produce a rapid (12–24 h) and significant increase in sediment stability that continued in a linear fashion over the period of the experimentation (228 h). Importantly, it was also found that light was required for the assemblages to produce this stabilisation effect and that removal of assemblage into darkness could lead to a partial reversal of the stabilisation. This was attributed to the breakdown of extracellular polymeric substances under anaerobic conditions. These data were supported by microelectrode profiling of oxygen and calcium. The structure of the assemblages as they formed was visualised by low-temperature scanning electron microscopy and confocal laser microscopy. These results have implications for the understanding of early stromatolite development and highlight the potential importance of the evolution of photosynthesis in the mat forming process. The evolution of photosynthesis may have provided an important advance for the niche construction activity of microbial systems and the formation and persistence of the stromatolites which came to dominate shallow coastal environments for 80% of the biotic history of the earth.

## Introduction

The drive to recognise the functional capabilities of diverse ecological systems is an emerging theme in modern ecology [1]. The impetus for this approach is related to the desire to assess the “ecosystem services” that habitats provide [2]. The first functional assemblages, or ecosystems, that can be recognised from the fossil record are the stromatolites which are often cited as the first indication of life on earth [3]. Stromatolites are laminated sedimentary structures formed by microbial activity [4] and are considered to have dominated the shallows of the Precambrian oceans. Stromatolites were the principle functional assemblages of the early earth and their structural and metabolic capabilities represented an early stage in the development of the highly-structured and self-organised systems that comprise modern microbial mats [5]. In terms of their ecological importance, there can hardly be a more significant “ecosystem service” than the production of oxygen and the consequent development of an oxygenic atmosphere, with the evolution of oxygenic photosynthesis among the cyanobacteria [6]. However, a second characteristic that is inherent in the development of modern stromatolites and microbial mats is the ability to trap and bind sediments [7]. The intricate metabolic cascades that have evolved between different components of the microbial assemblage that make up complex microbial mats depend on structural integrity to maintain the physicochemical gradients that drive the system [5], [6], [8]. The evolution of microbial mat systems is likely to have been influenced by the trapping and binding capacity of the constituent organisms. This leads to the identification of mat formers as “ecosystem engineers” [9]. However, although stromatolites are recognised as structures which are resistant to hydrodynamic forcing, there is very little information on the exact mechanisms of stabilisation and the way in which sediments may become bound. The debate often centres on the relative role of abiotic and biotic processes leading to particle capture and lithification. However, the arguments for and against the biotic influence should now be set aside by the recognition that stromatolites are not either solely biotic or mineral but the result of complex interactions between microbes, minerals and the environment [7].

The process of long-term stabilization in some stromatolites is mediated by the precipitation of calcium carbonate through microbially-mediated processes [3], [5], [10]. The relative role of precipitation and the biogenic processes that support it are also likely to have changed with the gradual diversification of life forms. It is recognised that we do not understand the nature of the early processes that led to the first accumulation of sediment and cells that led to stromatolite development [11]–[13]. There is no accurate model to represent this process today since the main players have changed with time and new forms have evolved, such as eukaryotes, which now also contribute to microbial mat formation. In addition, living stromatolites have become very rare as less ancient functional groups such as corals, bioturbators and grazers have emerged to compete with or exploit stromatolites [6]. However, living stromatolites do still persist in some limited areas [14] and this provides an opportunity for research into a modern analogue for these most ancient of ecosystems. The question of the early genesis of stromatolite forming assemblages cannot be addressed directly but information on the biostabilisation capacity of existing assemblages can be used to examine the potential of modern assemblages to trap and bind sediments. This paper presents the first measurements of the engineering capacity of natural stromatolite-forming assemblages under ambient conditions.

## Results

### Control systems

**Figure 1:**
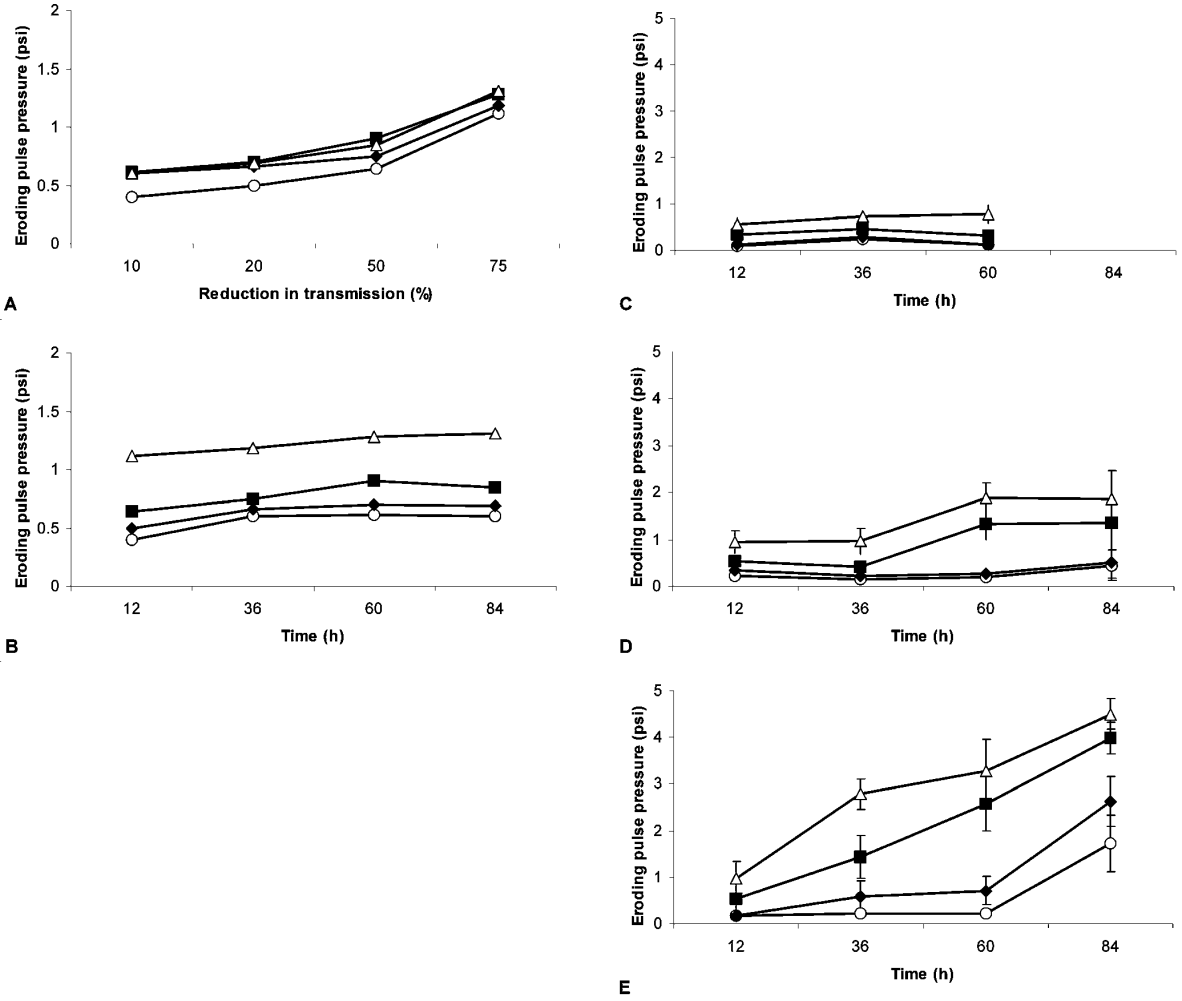
Erosion profiles from stromatolite material and controls measured during the winter. A. The mean pressure required to cause sequentially increasing levels of erosion in controls. Erosion thresholds (10, 20, 50 and 75% reduction in transmission against clear water [100%], respectively) were recorded for replicate incubations with increasing incubation time (o = 12 h, ♦ = 36 h, ▪ = 60 h and Δ = 84 h). B. Comparison of the mean pressure required to cause a specific level of erosion in control systems (o = 10%, ♦ = 20%, ▪ = 50% and Δ = 75%) with time. C–D. Comparison of the mean pressure required to cause a specific level of erosion for winter series (particle resuspension causing a reduction in transmission, o = 10%, ♦ = 20%, ▪ = 50% and Δ = 75%) against period of incubation for each of the experimental sites. C. Site 1. D. Site 5 and E. Site 10. (n = 7 for all treatments).

For each set of experiments a control comprised of carbonate sand (ooids) from beach areas among the stromatolites was examined (Figure 1A). The initial profile of erosion thresholds (10, 20, 50 and 75% reduction in water column transmission) after 12 h of incubation was significantly lower than the subsequent measurements (P<0.05, Nemenyi test, [15]. There was no further significant increase in stability with time. By examining the variation of each erosion threshold with time (Figure 1B), it was shown that the pulse pressure required to cause erosion after 12 h was the only value significantly lower than subsequent measures in the same series (H = 8.19 DF = 3 P = 0.042; H = 13.14 DF = 3 P = 0.004; H = 9.38 DF = 3 P = 0.025, for 10, 20 and 50% thresholds, respectively) with the exception of the maximum erosional threshold (75%) which showed no change with time (H = 1.16 DF = 3 P = 0.762)(Figure 1B).

### Experimental systems: Winter series

Columnar stromatolite material from site 1 showed little stabilization with time over the winter series (Figure 1C). There was a slight increase in force required to surpass the 10 and 20% erosion thresholds in the period between 12 and 36 h of incubation (H = 8.57 DF = 2 P = 0.014 and H = 9.56 DF = 2 P = 0.008) (Figure 1C) but no further statistical increase in stability. The ridge material from site 5 showed a greater variation in stability but little significant increase (Figure 1D). The final material for this series of analysis was derived from site 10. This site showed a clear and rapid increase in stability with time (Figure 1E). The force required to surpass the two most severe thresholds (50 and 75% reduction in transmission) increased in a linear fashion (Figure 1E). However, there was a significant increase in sediment stability using all measures (10% H = 15.12 DF = 3 P = 0.002; 20% H = 18.07 DF = 3 P = 0.000; 50% H = 19.18 DF = 3 P = 0.000; and 75% H = 18.38 DF = 3 P = 0.000).

### Experimental systems: Summer series

**Figure 2:**
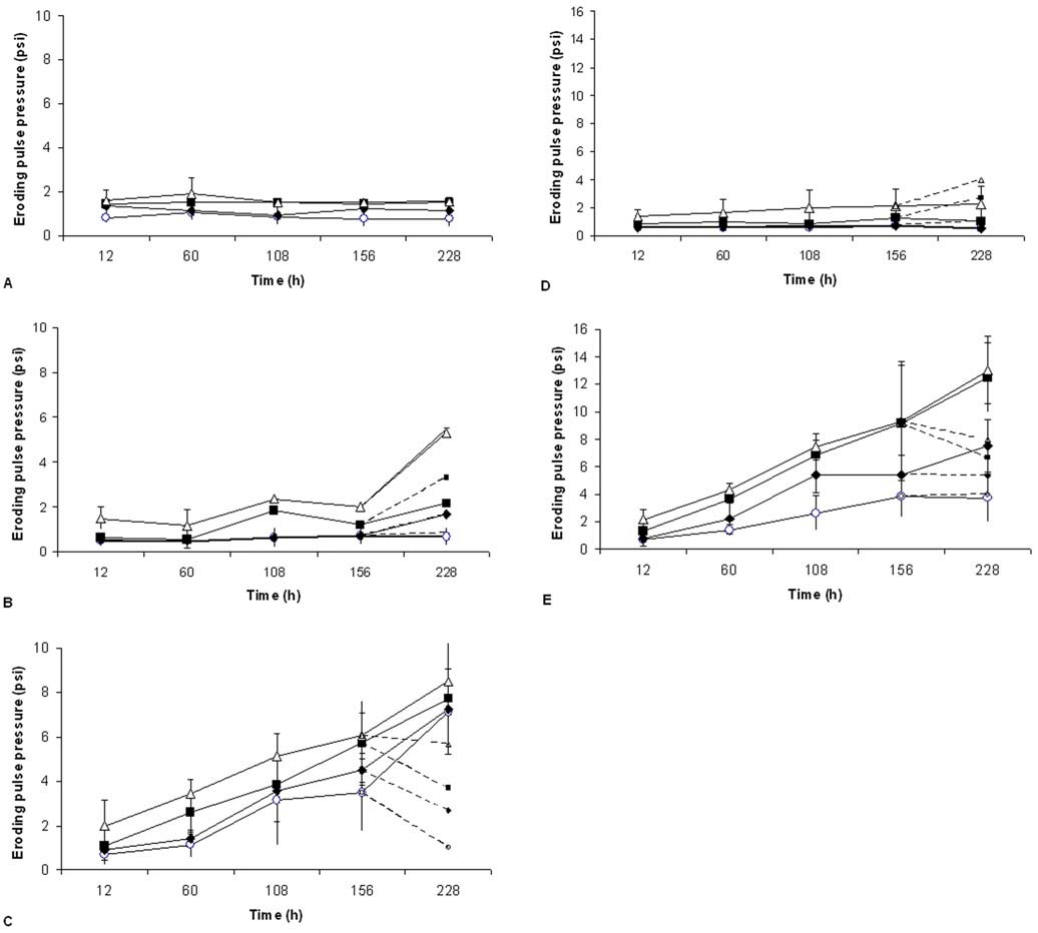
Erosion profiles from stromatolite material and controls measured during the summer. The mean pressure required to cause a specific level of erosion (particle resuspension causing a reduction in transmission, o = 10%, ♦ = 20%, ▪ = 50% and Δ = 75%, n = 7) against period of incubation for each of the experimental sites. A. Control of beach sand. B. Experimental replicates held in continuous darkness from site 1. For the penultimate incubation period, 3 replicates were transferred to the alternate condition (ambient light) as indicated by the dotted lines. C. Experimental replicates from site 1 kept under ambient light and temperature conditions. For the penultimate incubation period, 3 replicates were transferred to the alternate conditions (darkness) as indicated by the dotted lines. D. Experimental replicates from site 10 held in continuous darkness. For the penultimate incubation period, 3 replicates were transferred to the alternate condition (ambient light) as indicated by the dotted lines. E. Experimental replicates from site 10 kept under ambient light and temperature conditions. For the penultimate incubation period, 3 replicates were transferred to the alternate conditions (darkness) as indicated by the dotted lines. (n = 7 for all treatments except where stated).

A second series of experiments was conducted during the Bahamian summer (Figure 2A–C). A second control series was conducted and no significant increase in sediment stabilisation was found over an incubation period of 228 h (Figure 2A).

Material from sites 1 and 10 were selected as the extremes in response from the winter series of experiments and material was also incubated in darkness as well as under ambient light conditions.

The incubation of material from site 1, kept in darkness (Figure 2B), showed no significant increase in sediment stability with time for threshold 1 (10%). However, the higher thresholds did show increases in stability but only in that the final measurement was significantly higher than previous measurements (20%; H = 13.99 DF = 4 P = 0.007: 50%; H = 13.29 DF = 4: 75%; H = 14.24 DF = 4 P = 0.007). During the final stages of the test, 3 replicate systems were transferred into ambient light conditions (dotted lines on Figure 2B). This had no significant effect in terms of stability (all P>0.05). For incubation under ambient light, the pattern was quite different with a significant linear increase in sediment stability with time for all erosion thresholds (H>19 DF = 4 and P≤0.001 in all cases) (Figure 2C). After 156 h of incubation, 3 of the seven replicate systems were moved into continual darkness (dotted lines, Figure 2C). The stability in these systems decreased in comparison to the 156 h mark (Figure 2C). This decline was assessed against equivalent replicate systems kept under ambient light and shown to be significant (Man Whitney Test, P<0.001).

This work was repeated for material from site 10 (Figure 2D–E). Material cultured in darkness showed no statistical increase in stability with time (Figure 2D). Where replicates were transferred from darkness into the light there was a noticeable increase in stability but these differences were not significant. The stability of systems kept under ambient light increased in a linear fashion (Figure 2E) (P<0.001). The slope of the increase was slightly different among the levels of erosions threshold with the more sensitive thresholds (10 and 20%), increasing more slowly than the more extreme thresholds (50 and 75%). After 156 h of incubation, 3 of the seven replicate systems were moved into continual darkness (dotted lines, Figure 2E). The stability of the 3rd and 4th erosion thresholds in these systems decreased in comparison to the 156 h mark (Figure 2E). This decline was assessed against equivalent replicate systems kept under ambient light and shown to be significant (Man Whitney Test, P<0.001).

### Calcium micro-profiles: Summer series

**Figure 3:**
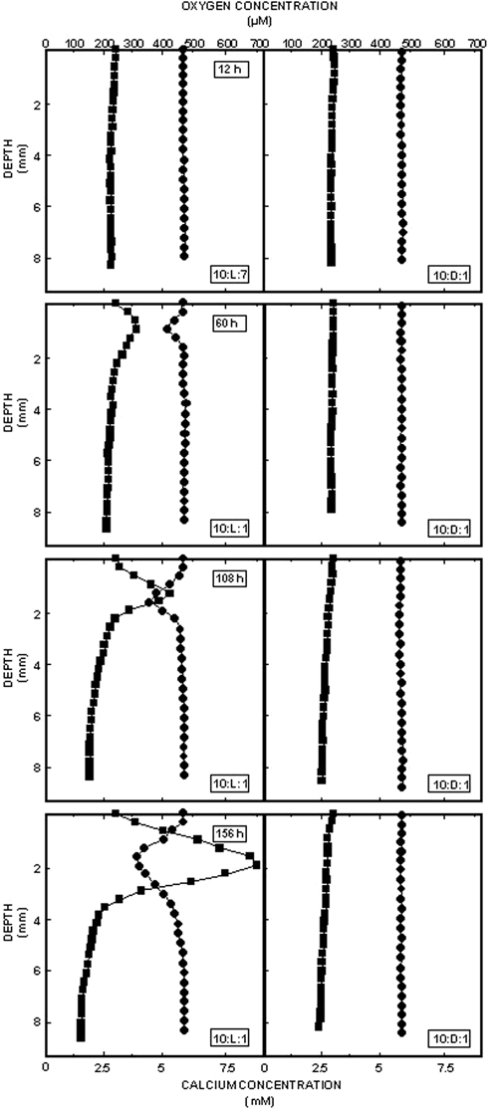
Oxygen and calcium concentrations within the mat systems. Depth profiles of oxygen (▪) and calcium (•) in the upper 8 mm of the sediments from site 10. Left hand side panels depict light incubations; right hand side panels represent dark incubations. Each profile corresponds to the average of three measurements. From top to bottom, measurements were taken after 12 h, 60 h, 108 h and 156 h, respectively. Average light intensities were 1613, 1241,1976, and 1846 µE m^−2^ s^−1^, during the 12 h, 60 h, 108 h, and 156 h afternoon O_2_ measurements, respectively.

Preliminary measurements conducted during the winter series indicated the potential of the reconstituted mats to rapidly engage in calcium binding (data not shown). During the summer series, profiles for O_2_ and Ca^2+^ were measured throughout the experiment (228 h). Representative profiles for the light/dark and dark incubations during the initial 156 h of the experiment (average of three measurements, top 6–8 mm depicted only; Figure 3) showed that dark incubations resulted in typical diffusion profiles of O_2_ and Ca^2+^ (Figure 3: right hand panel). From the early stages of mat development, there was a potential to bind Ca^2+^ from the overlying water as the minimum in the profile of this suggested. Removal of Ca^2+^ is a critical step in CaCO_3_ precipitation[3]. After 60 h of incubation, ca. 0.9 mM calcium (15%) was bound at the depth where the O_2_ maximum was found. This increased to 1.2 mM Ca bound (20%) after 108 h, while after 156 h of incubation, ca. 2.1 mM of calcium (38% of total) was bound in the zone of maximum O_2_ production. Measurements taken after 228 h (not shown) revealed that 2.0 mM was bound, indicating that the maximum calcium binding capacity was reached after 156 h. In the experiments where ambient light treatments were transferred to continuous darkness, the oxygen peak disappeared and the calcium minimum decreased in magnitude (data not shown). In contrast to the dark incubations, ambient light/dark cycles supported a rapid mat formation (Figure 3: left hand site panels): at ca. 1–2 mm depth an O_2_ peak, characteristic for mats including intact stromatolites [16] was observed. The oxygen maximum migrated from 0.75 mm after 60 h (ca. 140% O_2_ saturation at peak) when it was first observed, to 2.0 mm after 156 h of incubation in the natural light-dark cycle. Towards the end of the experiment, peak values in excess of 300% O_2_ saturation were observed (Figure 3, bottom left panel).

### Imagery of stromatolite systems

**Figure 4:**
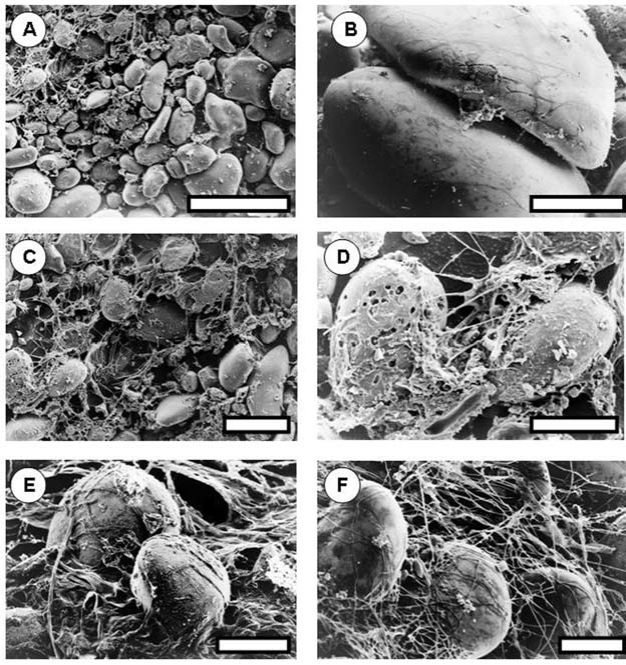
Low-temperature scanning electron micrographs of reconstituted stromatolite material. A. Material after the first 2 days of incubation. Surface ooids and organic material. B. Organic linkages between ooid grains. C. Further development of organic material at the surface. D. Detail of ooids showing beginning of a complex matrix of polymers and cyanobacterial (Schizothrix) filaments. E–F. The cyanobacterial matrix becomes denser eventually enveloping the ooid grains. Bar markers: A = 800 um, B = 100 um, C = 400 um, D–F = 100 um.

**Figure 5:**
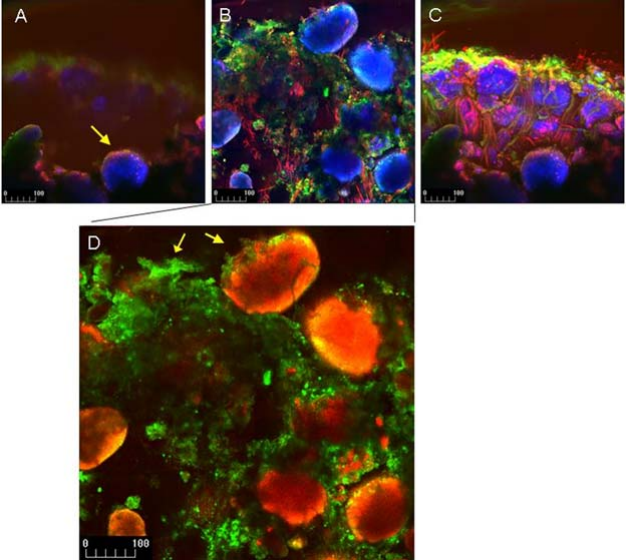
CSLM images showing the initial trapping of ooids on mat surface. A–C. The accumulation of EPS and abundant filamentous cyanobacterial cells that begin to surround ooids to form a structured microbial community. Note the autofluorescence and scattering of aragonite (blue), cyanobacterial pigment autofluorescence (red) and heterotrophic cell clusters (green). D. Sediment ooids appear orange, while EPS stained with lectin Con-A appears green. (Scale bar given in um).

Low-temperatures scanning electron micrographs of the re-constituted stromatolite material provided qualitative evidence of the nature of the binding mechanism (Figure 4). Stages in the binding of the surface ooids were observed. Binding was achieved by a surface matrix of cyanobacterial filaments (Figure 4A–C). Organic material was also found closely associated with the ooids (Figure 4C and D). Fracturing of random ooids revealed micro-boring organisms including the cyanobacterial species, *Solentia* sp (Figure 4D). This was supported by observation of the developing mat system using confocal laser microscopy (Figure 5). The CSLM imagery showed the accumulation of EPS associated with the surface of the ooids (Figure 5A). The further development of EPS binding and physical trapping by cyanobacterial filaments was clearly demonstrated (Figure 5A–C).

### Comparison between systems

**Figure 6:**
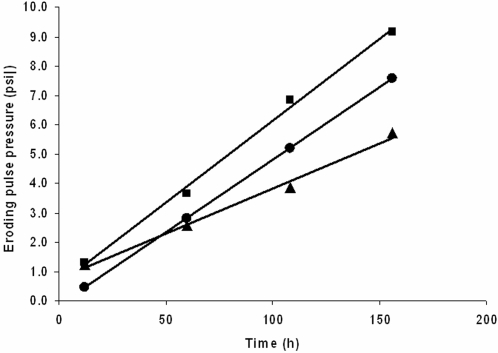
Linear relationship between eroding pressure and time. Linear regression lines for the equations given in Table 1. The relationships represent the increase in stability with time for the winter series from site 10 (•), the summer series from site 1 (▴) and the summer series from site 10 (▪). For clarity, the mean values of the data groups are indicated by the symbols however, the regressions were calculated using all data points.

Comparison between seasons and sites (1 and 10) was conducted by selecting and comparing a single erosion threshold (50%) between treatments. The 50% threshold showed a linear increase in stability over the incubation period for all systems with the exception of the winter series at site 1 (Figure 1C) in which there was no significant increase (df 20, r^2^<0.00, p = 0.89). For the other comparisons, the linear regression data for each is given in Table 1 and graphical relationships in Figure 6. The slope of all lines proved to be significantly different (P<0.01). Site 10 showed a more rapid rate of stabilisation than site 1 under both summer and winter conditions and in each case the systems were increasing in stability over the entire course of the incubation. Site 1 showed no increase in stability during the winter but showed significant stabilisation in the summer.

**Table 1:** Regression analysis of the 50% erosion threshold of three experimental trials.

Trial	Equation	R^2^ (adj)	ANOVA		
			DF	F	P
Winter 10	PSI = −0.155+0.0496 h	79.5	26	101.79	0.000
Summer 1	PSI = 0.752+0.0308 h	51.1	26	29.5	0.000
Summer 10	PSI = 0.545+0.0558 h	66.3	26	54.13	0.000

## Discussion

### Light enhanced stromatolite binding

Re-constituted stromatolite material shows a clear capacity to re-establish a stabilised substratum. For the first time, the rate of sediment stabilisation and engineering capacity of the microbial assemblages that comprise living stromatolite has been shown in an experimental study. Stabilisation of microbial systems as compared with controls began within hours and were still increasing in stability over the entire course of the incubation (228 h). There was a variation in results from different sites and variation between seasons, with sites 1 and 10 more active in the summer and the microbial populations of site 10 being more effective stabilisers than those from site 1. Additional microbiological investigation would be worthwhile to establish the variation in assemblage composition. Site 1 did not produce effective stabilisation during the winter (Figure 2A). In all cases, light was a critical factor in the rapid development of cohesion within the systems. There was some indication that stabilisation might be possible in darkness (Figure 2B) but only after extended incubation and to a much lesser extent than found with illuminated systems. Further, there was an observed decrease in sediment stabilization of light-induced mats which were transferred to darkness (Figure 2C). This might have occurred due to heterotrophic degradation of photosynthetically-derived EPS. This information opens a new possibility in the interpretation of ancient stromatolite material. If modern stromatolites provide a reasonable analogue for their ancient ancestral forms [17], then we might conclude that the initial biogenic stabilisation of sediments by stromatolites probably became more rapid and effective after the evolution of photosynthesis. This seems sensible since the processes of biogenic stabilisation are often associated with the production of organic molecules secreted as a by-product of photosynthesis. These molecules provide a large proportion of the extra cellular polymeric substances found in modern surficial sediments [18] and stromatolites [19]. EPS is often cited as one of the major mechanisms of biogenic stabilisation [20]. The capacity of extra-cellular organic substances to stabilise sediments has been widely demonstrated in laboratory and field studies [21], [22]. This suggests that early biofilms formations which pre-dated the evolution of photosynthesis might be more transient and delicate than later forms, in keeping with the slow development of stability in the dark incubation in the present study. However, the metabolic process carried out by non-photosynthetic bacteria may still have been influential on the formation of carbonates and evaporites [13], [23]–[26]. The advent of photosynthesis and the capacity of biofilms to produce organic molecules is likely to have worked in tandem with existing non-photosynthetic organisms increasing the likelihood of stromatolite formation. The role of the varied organic molecules associated with biofilms, microbial mats and stromatolites is continuingly being investigated and expanded [18], [19].

### The mechanistic nature of binding

The present study suggests that stromatolite assemblages are capable of rapid and effective stabilisation of suitable substrata. The production of extra cellular polymeric substances certainly plays a role in the stabilisation of biofilms and microbial mats as highlighted above (Figure 4). In addition, the filamentous nature of the cyanobacteria appears to become an effective stabilising mechanism (Figures 4 and 5). The evolution of a filamentous growth habit may also have been important to enhance the potential for stable biofilm formation and some workers have noted that in evolutionary and morphological terms cyanobacteria have changed little since their first preservation in the fossil record [17]. The lack of stabilisation in systems deprived of light may be directly as a consequence of the lack of photosynthesis and hence organic exudates or may also be a secondary effect mediated by the lack of a migrational cue for cyanobacteria to accumulate at the sediment surface. However, it is likely that both processes will influence the onset of surface cohesion. Variation in the trapping and binding capacity of stromatolite assemblages has been suggested to influence the nature of the mineralization process and hence lamination structure [10].

### The lithification process

The initial biogenic stabilization of depositional systems may well be a requirement to allow or enhance future lithification of the sediments. Reworking of the matrix by wind, waves or tides is unlikely to be conducive to stromatolite preservation. Initial analysis of the stromatolites as the assemblages reorganized after homogenization showed that the initial stabilization process was independent of calcium concentration, but likely due to the concentration of biomass and associated EPS. This process is probably driven by light induced cyanobacterial movement [27]. These cyanobacteria are major producers of EPS [6], [28], which effectively scavenges calcium [3]. After 156 h of incubation, the binding sites in the EPS matrix are saturated with calcium, and calcium carbonate precipitation is likely to commence as soon as the geochemical conditions allow this. In the initial stages of mat development, as seen towards the end of the present experiments, this precipitation is enhanced by EPS degradation (microbial or via UV decay) in combination with an elevation of the pH, which is found during maximum photosynthesis during the afternoon [16]. The rapid binding of calcium and early saturation of the EPS matrix with this material may be surprising, but is also an absolute requirement for early mats to survive the extreme hydrodynamic conditions that prevail at the Highborne Cay site. Where illuminated systems were transferred to darkness, part of the EPS matrix may have been degraded, releasing the calcium. This corroborates the observation of loss of stability (lowering of the erosion threshold) during this treatment as described above. Controls (ooids only) showed virtually vertical depth profiles that did not change over the course of the experiment (not shown).

### Limitation and questions

The artificial homogenisation of stromatolite material is a major and unusual disturbance. Even after this rather brutal treatment the microbial assemblage is capable of re-stabilisation despite have sustained considerable damage and dispersal among a far greater volume of sediment than is normal for the highly stratified natural assemblages. The sharp gradients that exist in the stromatolite will have been destroyed but begin forming again as soon as the material is allowed to settle. Additionally, although the homogenisation is extreme, the effects of Hurricane Rita did lead to the fragmentation and dispersal of stromatolite material in a somewhat similar manner to the current experiment (Reid pers comm.). However, the veracity of the experimental treatment was not the issue here. What is more significant is that the stromatolite assemblages have proven themselves to be rapid and highly effective ecosystem engineers. These systems are the nearest analogue we have to the ancient microbial mats that were arguably the first organized ecosystem on the surface of the planet. The evolution of photosynthesis may have provided an important advance for the niche construction activity of microbial systems and the formation of the stromatolites which came to dominate shallow coastal environments for 80% of the biotic history of the earth [19].

## Materials and Methods

### Sites

**Figure 7:**
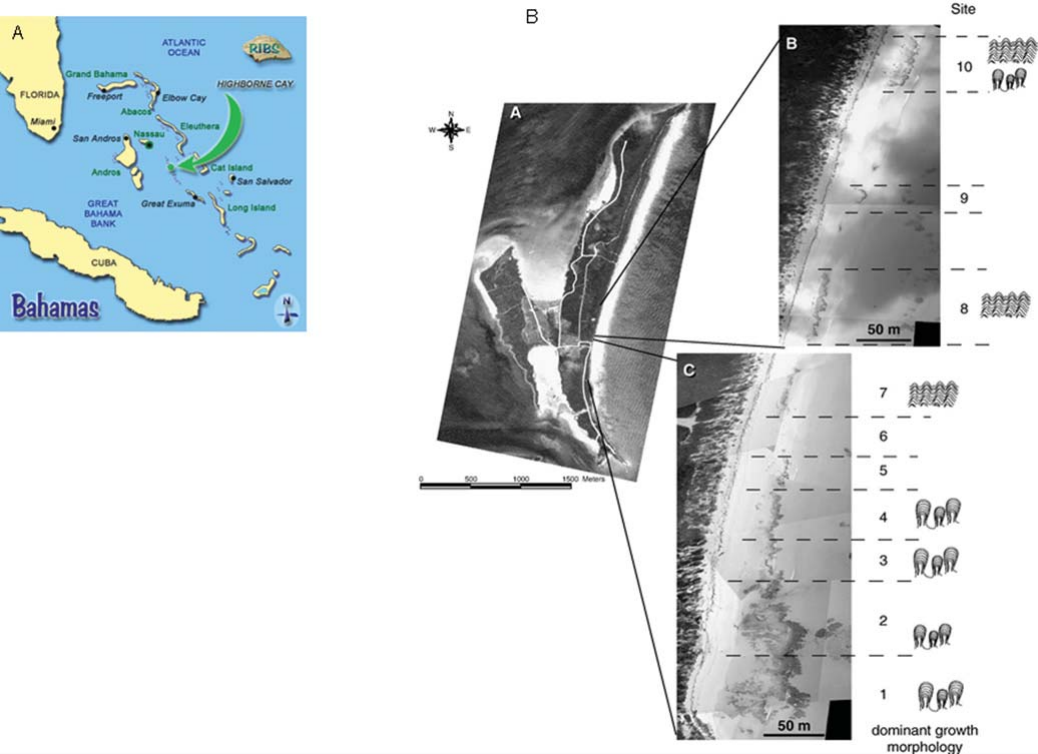
Highborne Cay in the Bahamas. The location of Highborne Cay in the Exuma chain of Bahamian Islands. A. Aerial detail of Highborne Cay. B. The samples sites from the North-easterly beach of the island as described previously [29].

The material for study was obtained from shallow to sub-tidal regions of Highborne Cay (76°49′W, 24°43′N), Exuma Chain of Islands in the Bahamas (Figure 7). The morphology and extent of the stromatolite reef system is described in detail by Andres and Reid [29]. Two major morphologies of stromatolite were described, columnar stromatolites and stromatolite ridges. Stromatolite material was collected from three sites along the beach (Figure 7B). The sites corresponded with areas under investigation through the “Research Initiative on Bahamian Stromatolites” (RIBS) project (http://www.home.duq.edu/stolz/RIBS/index.html). Material was collected on two RIBS cruises, the first in November 2003 (winter series) and the second in July 2004 (summer series).

### Preparation of sediments

Samples of stromatolite from the sample areas were collected and taken to the research vessel laboratory (the RV Walton-Smith). The living stromatolite material was gently broken down by hand into constituent grains and passed through a 1 mm sieve to remove large fragments but retain the carbonate ooid grains and associated microbial populations. The reconstituted material was placed in small square trays (15×15×10 cm) on a base of beach ooid material (3 cm deep) to form a 2 cm deep layer, shaken gently to smooth the surface of the reconstituted bed and placed in an open outdoor aquaria supplied with running seawater under a natural day/night cycle. The systems were maintained for a maximum of 228 h. Experimental runs were conducted on two RV Walton Smith cruises (November 2003 and July 2004). In the first series of experiments under winter conditions (11/03), material from sites 1 (columnar), 5 (ridge) and 10 (ridge) was collected (Figure 7). Samples were maintained under natural light conditions. In the following summer series (7/04), material was collected from RIBS sites 1 (columnar) and 10 (ridge) and, in addition to ambient day/night cycles (n = 7), replicates were also kept under the condition of continuous darkness (dark treatment, n = 7). In all experimental runs, control systems were established using natural beach carbonate sand from among the stromatolite heads (n = 7 for all initial experiments).

### Sediment stability

The stability of the surface was measured using a Cohesive Strength Meter [30], [31]. During each erosion test, 4 relative measures of erosion were recorded related to the resuspension of ooids from the surface of the bed. Data are presented as a % loss in transmission against clear water (100%).

1. Erosion threshold 1: 10% decline in transmission
2. Erosion threshold 2: 20% decline in transmission
3. Erosion threshold 3: 50% decline in transmission
4. Erosion threshold 4: 75% decline in transmission

These thresholds represented the first unequivocal reducing in transmission (1), the general erosion of surface material (2), the erosion of underlying material (3) and general bulk erosion (4). These thresholds were established by observation on the influence of the jet on the preliminary tests of reconstituted stromatolite material.

### Oxygen and calcium microprofiles

Depth profiles of O_2_ and Ca^2+^ of the upper 15 mm of the sediments were measured to determine microbial activity and location and also the potential of the reconstituted system (i.e., biomass and exopolymeric substances, EPS) to bind calcium [16]. Glass microelectrodes with a tip diameter of less than 100 µm (Unisense, Aarhus, Denmark; Diamond General, Ann Arbor, MI, USA) were deployed using a motor driven micromanipulator (National Aperture, NH, USA) in combination with a picoammeter (Unisense) and high-impedance millivolt meter (Microscale Measurements, The Hague, The Netherlands), for O_2_ and Ca^2+^, respectively. Measurements were made in samples from locations 1 and 10, as well as in controls (beach ooids), under ambient light conditions for light treatments and in the shade for dark treatments. The light intensity was recorded with a LiCor LI 250A meter equipped with a quantum sensor (LiCor, Lincoln, NE, USA). At each time point (12, 60, 108, 156 h), three replicate measurements were taken and average values calculated before generating depth profiles. These measurements were continued for a further 72 h (to a total experimental time period of 228 h).

### Low- temperature scanning electron microscopy

The microstructure of selected samples was examined by low-temperature scanning electron microscopy (LTSEM) after Paterson [32]. Samples were taken using small plastic cores (3 cm id) and quench frozen in liquid nitrogen (LN_2_). The samples were transported in dry ice and then stored at −80°C. Before examination, samples were transferred back into LN_2_. The frozen material was fractured under LN_2_ and mounted on a specialized mechanical stub and introduce to the cold-stage of a scanning electron microscope (JEOL 35CF SEM fitted with Cryo capability, Oxford systems). Surface water was removed by sublimation into vacuum (−90°C) and the samples coated with gold and examined while still frozen (−180°C).

### Confocal Scanning Laser Microscopy (CSLM)

Imaging by confocal scanning laser microscopy (CSLM) was conducted using a Zeiss LSM 510 Meta Confocal system, equipped with Zeiss Axioplan 200 motorized microscope and a 405 diode argon, red and green He/Ne lasers. Image resolution was 512×512 pixels [33].

### Statistical analysis

Stability data was not normally distributed therefore the non-parameteric Kruskal Wallace test was applied [15] using statistical software (Minitab). Seven replicates were maintained in all cases with the exception of the summer series where after the penultimate measurement (156 h incubation) 3 replicates were transferred to the alternate condition (from ambient light to darkness or vice versa). Where the Kruskal Wallace was significant for a difference among groups, the test was augmented by a *post-hoc* multiple comparison to identify the different groups [15]. In the few cases where the comparison was unbalanced (containing a group of n = 4) the procedure of Dunn 1964 [15], was applied. Where simple pair-wise comparisons were made, a Man Whitney test was employed.
